# The severity of ulnar variance compared with contralateral hand: its significance on postoperative wrist function in patients with distal radius fracture

**DOI:** 10.1038/s41598-018-36616-5

**Published:** 2019-02-18

**Authors:** Xu Jianda, Yuxing Qu, Li Huan, Qian Chen, Chong Zheng, Wang Bin, Shen Pengfei

**Affiliations:** 1 Department of Orthopaedics, Changzhou Traditional Chinese Medicine hospital, Changzhou, 213003 China; 2 Department of Arthroplasty, The First People’s Hospital of Changzhou, Changzhou, 213003 China; 3 Department of Orthopaedics, Changzhou Wujin People’s Hospital, Changzhou, 213003 China

## Abstract

The aim of this study was to detect the severity of ulnar variance (UV) compared with contralateral hand on postoperative wrist function in patients with distal radius fracture. 116 cases with unilateral distal radius fracture were retrospectively analyzed and divided into high or low UV severity groups (Dividing value = 2.5 mm). The following parameters were used to evaluate the effect: palmar tilt, radial inclination, VAS score, DASH score and wrist function. The severity of UV existed widely, accounting for 93.1% (108 cases). The severity of UV correlated with palmar tilt, radial inclination, grip strength, VAS score, DASH score and the wrist function (P < 0.05). Log-rank analysis showed that the severity of UV, palmar tilt, radial inclination were important factors influencing the joint function postoperatively (P < 0.0 5). Multivariate analysis confirmed that the severity of UV was an independent and significant factor on wrist function (P = 0.010). And the palmar tilt was also an important factor influencing wrist function (P = 0.047). The severity of ulnar variance compared with contralateral hand is an independent and significant factor on wrist function, which should be considered as an important step during preoperative plan.

## Introduction

The distal radius fractures are the most common upper extremity fractures, accounting for about 16%^1^. Most of them destroyed the anatomy of distal radioulnar joint and antibrachio-carpal joint, resulting in severe chronic pain, wrist stiffness, grip strength weakening and dysfunction^2^. In latest years, most newly surgical methods (percutaneous Kirschner wire fixation, external fixation and open reduction and internal fixation) are used^3^, a certain adverse prognosis rate is still not uncommon^4^.

Previous studies had shown that the change in ulnar variance (UV) up to 2.5 mm usually resulted in a significant change from the normal biomechanics of wrist, which brought higher risk of severe wrist pain^5^. Jianli B used 11 fresh cadaver specimens to investigate the effect of radial shortening on wrist joint mechanics, and confirmed that the ulnar variance affected the wrist function. The transmitted load distribution between the radius and ulna was measured. The negative ulnar variance brought 94% load transmission in radial side and 6% in the ulnar side, while positive ulnar variance with 69% load transmission in radial side and 31% of the ulnar side^6^. Tomaino MM found that ulnar impaction syndrome occurred not only in ulnar positive wrist, but also in wrists with either ulnar negative or neutral variance^7^. All these reminded us that individual change of UV might be an important prognostic factor.

To our knowledge, there is little study about the severity of UV affecting the clinical prognosis in patients with distal radius fracture. We hypothesized that the severity of UV was an important prognostic factor affecting wrist function. The aim of this study was to detect the severity of UV compared with contralateral hand on postoperative wrist function.

## Materials and Methods

This clinical trial was designed and approved by the Ethics Review Committee of Changzhou Traditional Chinese Medical Hospital, affiliated to Nanjing University of Traditional Chinese Medicine. All methods were performed in accordance with the relevant guidelines and regulations. Written informed consent was obtained.

From Jul. 2010 to Jul. 2015, 116 cases, ASA I-III, aged 19–68 (average, 46.8 ± 14.7) years, unilateral distal radius fractures were retrospectively analyzed. All these patients (42 males and 74 females) enrolled were recommended to undergo open reduction and internal fixation by palmar approach. Of them, 64 patients got surgical treatment within 48 hours after trauma, and 52 cases in 3–8 (5.7 + 2.1) days after trauma. According to Femandes type^8^, 13 of type I, 63 of type II, 30 of type III and 10 of type IV. The inclusion criteria were over 18 years old; underwent open reduction and internal fixation of unilateral distal radius fracture; no disorders affecting the healthy side upper limb function; no obvious mental disorders and could accomplish a complete clinical follow-up. Patients were excluded if they had disorders affecting contralateral limb function disorder or older than 18 years; if they had a history of trauma to contralateral hand in the past years. According to the variance of UV (Dividing value = 2.5 mm^5^), all enrolled patients were divided into high UV severity group (45 cases) and low UV severity group (71 cases).

The operations were performed by one senior experienced orthopaedic surgeon major in upper limb fracture. Mobilization started from the second day postoperatively with the same rehabilitation scheme. On the following days, patients attended rehabilitation twice a day.

### Study clinical parameters

All patients got followed-up at 1, 3, 6, 12 months postoperatively. Follow-up duration was defined as the date of operation to the date of latest follow-up. Pre-operative assessment involved bilateral wrist standard radiographs. Post-operative assessments involved radiographic evaluations (including ulnar variance, palmar tilt, ulnar variance) and clinical evaluations (including grip strength (Jamar, New York), joint function, visual analogue scales (VAS) score and the Disabilities of the Arm, Shoulder, and Hand (DASH) test).

### Radiographic evaluation

Follow-up radiographs were assessed for fracture congruity, restoration of radial inclination and palmar tilt. Fracture union was established radiographically by the presence of bone continuity or bridging callus on both the posteroanterior and lateral views associated with no signs of implant loosening^9^.

Ulnar variance was measured in the standard posteroanterior x-ray of the wrist. A line, perpendicular to the long axis of the bone, was drawn through the most ulnar point of distal articular surface of the radius. The distance between this line and the ulnar dome in millimeters was defined as ulnar variance^10^. The data analysis was independently evaluated by two different radiologists using PACS system. The final results were the mean value.

Severity of UV = post-operative UV − contralateral UV

The positive indicated the UV increased compared with the contralateral side, while the negative with decreased variance.

### Clinical evaluation

Wrist joint mobility

Wrist joint mobility was measured using a goniometer^11^.

Visual analogue scales(VAS)^12^

All patients were asked to mark their level of pain on a 10 cm visual analogue scale. Score 0 represented no pain, while 10 represented the worst pain.

Grip strength was measured using a calibrated hydraulic hand dynamometer (Jamar, New York) and compared with the contralateral wrist.

Disabilities of the Arm, Shoulder, and Hand (DASH) test

The DASH test was a self quantified questionnaire to evaluate the upper extremity symptoms and the ability to engage in daily activities, including 30 projects such as physiological activity, severity of symptoms, damages on the social activities and so on. The DASH score less than 15 was regarded as fine^13^.

### Statistical analysis

The SPSS statistical package (Version 17.0, SPSS Inc., Chicago, IL, USA) was employed for statistical analyses. The normality of distribution for continuous numeric variables was assessed by Kolmogorov-Smirnov test. According to normally distributed or not, the variables were presented as means with SD, and otherwise as medians with inter-quartile ranges (95% confidence intervals, 95% CI). Student’s t-test for normally distributed continuous variables, while others using Pearson’s χ^2^ test. Associations between prognostic variables and excellent rate were analyzed by log rank test. Multivariate analysis employed Cox’s proportional hazards regression model with a forward conditional stepwise procedure to determine the acting independent factor. The positive statistical significance was set at P < 0.05.

## Results

All 116 patients got bone united uneventfully, and no secondary displacement, superficial or deep infection was found. The average time to clinical and radiographic union was 8.1 ± 2.3 weeks (range, 6–11 weeks). The patients resumed their previous work at an average of 12 ± 3.6 weeks (range, 8–16 weeks) post-operatively. The total average DASH score was 9.7 ± 5.6, representing high level of satisfaction.

### The relationship between the severity of UV and clinical characteristics

The severity of UV existed widely in the patients with distal radius fracture, accounting for 93.1% (108 cases). Of them, 100% in high UV variance group and 88.73% in low UV variance group.

No significant correlation was found in age, gender, injury side, fracture type, surgical preparation time and bone graft or not (P > 0.05, Table 1). The severity of UV correlated with palmar tilt, radial inclination, grip strength, VAS score, DASH score and the wrist function (P < 0.05, Table 1).

**Table 1:** The relationship between the severity of UV and clinical characteristics.

		High UV severity group	Low UV severity group	*P*-Value
Age(u)		47.2 ± 18.7	44.6 ± 20.5	0.102
Gender	male	15	27	0.608
	female	30	44	0.993
Injury side	left	21	28	0.449
	right	24	43	
Fracture type	I	7	6	0.632
	II	23	40	
	III	12	18	
	IV	3	7	
Surgical preparation time	≤48 h	26	38	0.399
	>48 h	19	33	
Bone graft	yes	11	19	0.479
	no	34	52	
Flexion (°)		50.1 ± 11.7	52.4 ± 10.3	0.037
Extension (°)		53.5 ± 12.2	56.2 ± 11.5	0.049
radial inclination (°)		28.8 ± 9.9	31.7 ± 10.3	0.041
radial deviation (°)		21.1 ± 8.3	23.6 ± 7.2	0.033
Pronation (°)		73.7 ± 9.3	77.5 ± 8.9	0.026
Supination (°)		75.4 ± 12.4	80.8 ± 11.2	0.032
Palmar tilt (°)		13.7 ± 3.5	15.3 ± 2.8	0.041
radial inclination (°)		22.3 ± 6.8	20.7 ± 7.9	0.049
Grip strength, (Kg)		26.9 ± 8.7	31.5 ± 8.1	0.027
VAS		3.1 ± 1.6	1.4 ± 1.1	0.013
DASH		23.5 ± 10.4	12.8 ± 7.6	0.002

### The prognostic factors and joint function postoperatively

Log-rank analysis showed that the severity of UV, palmar tilt, radial inclination were important factors influencing the wrist joint function postoperatively (P <0.0 5, Table 2). No significant correlation was found in age, gender, injury side, fracture type, surgical preparation time and bone grafting or not (P > 0.05, Table 2).

**Table 2:** Log-rank analysis of prognostic factors in patients with distal radius fractures.

	DASH excellent rate (%)	*P*-Value
All patients	92.2	
Age (≤50/>50,y)	94.3/89.1	0.310
Gender (male/female)	92.9/91.9	0.185
Injury side (left/right)	93.9/83.3	0.164
Fracture type (I/II/III/IV)	92.3/96.8/86.7/80.0	0.155
Bone grafting (yes/no)	86.7/94.2	0.234
surgical preparation time (≤48 h/>48 h)	90.6/94.2	0.359
palmar tilt (≤14/>14°)	81.8/95.2	0.030
radial inclination (≤22/>22°)	95.1/84.8	0.078

### Multivariate analysis of prognostic factors in patients with distal radius fractures

Multivariate analysis confirmed that the severity of UV was an independent and significant factor on wrist function (P = 0.010, Table 3). And the palmar tilt was also an important factor influencing wrist function (P = 0.047, Table 3).

**Table 3:** Multivariate analysis of prognostic factors in patients with distal radius fractures.

Variable	B-value	Total excellent rate (%)		
		Hazard ratio	95% confidence interval	*P*-Value
The severity of UV	2.421	0.473	0.226–0.686	0.010
Palmar tilt	1.467	0.927	0.431–0.723	0.047

## Discussion

The importance of ulna variance was confirmed and considered as a main prognostic factor for instability in distal radial fractures^14^. Just 1 mm change usually brought an increase in the mechanical load through the ulna by more than 25%. Ulnar shortening osteotomy was widely used in mal-united distal radius fractures, and a 7-year follow-up found that negative UV usually meant a worse wrist function^15^. The re-established UV usually maintained the cartilage surface at the distal ulna and tightened the ulno carpal ligamentous complex^16^.

However, the UV is not unchangeable in all human beings. Nathan PA *et al*. had confirmed that varying degrees of ulnar-minus variance were found in about 25% normal wrists^17^. Nakamura and coworkers found that UV correlated positively with age in normal wrists, but not in patients with Kienböck’s diseases^18^. Yoshida and colleagues compared the mean UV in patients with or without Kienböck’s disease, and found that more positive mean UV occurred in the older control subjects compared with the younger control subjects, especially for women^19^. Gelberman RH *et al*. found that more positive UV occurred in blacks compared with whites after measuring the UV without Kienböck’s disease^20^. In our study, the variance of UV existed widely in the patients with distal radius fractures, accounting for 93.1% (108 cases). All these reminded us that individual change of UV might be an important prognostic factor.

In present study, the severity of UV was calculated compared with contralateral hand, which commendably avoided individual variation. Multivariate analysis confirmed that the variance of UV was an independent and significant factor on wrist function (P = 0.010). At the same time, the study also found that the palmar tilt was an important factor influencing wrist function (P = 0.047). Tahririan MA pointed out that in patients with closed reduction, radial shortening of more than 6.5 mm, loss of radial inclination of more than 6.5 degrees and age above 52 were the most important predictive factors for in stability^21^. These results just identified the main predictive factors on secondary displacement in distal radius fractures, but the effect of radial inclination on the functional prognosis was not confirmed. Stahl S and Goeminne S had concluded that negative UV negatively correlated with wrist function in Kienbock diseases^22,23^. Kodama N *et al*. had concluded the same results with our study. They assessed the radiographic findings of patients with no wrist dysfunction and revealed that palmar tilt and UV were the main prognostic factors for clinical outcomes. These parameters usually brought worsen outcomes when exceeding a tolerable range^24^.

A number of reports have provided the importance of radiographic parameters on wrist function, but no data in the literature concerning accurate association between different parameters. Stephen A. Brennan *et al*.^25^ compared the radiographic and functional outcomes of 318 patients with distal radius fractures. And they found higher values for radial inclination, radial length and volar tilt correlated with better functional outcome as measured by disabilities of the arm shoulder and hand (DASH) and patient rated wrist evaluation (PRWE) scores. Lower values for ulnar variance correlated with better functional outcome. However, the relationship between ulnar variance and other radiographic parameters wasn’t confirmed. P Dario *et al*.^26^ evaluated 51 patients treated with volar locked plate for articular unstable distal radius fractures and found that ulnar variance and volar tilt are the most important radiographic parameters influencing the final functional outcome. Kodama *et al*.^24^ reported similar results: volar tilt and ulnar variance were significantly correlated with clinical outcomes, although radius height was not evaluated. In our study, the severity of UV and palmar tilt were also the important parameters affecting function.

This study has some inherent limitations. It should be pointed out that the sample size enrolled is relatively small, although illustrating the prognostic value of severity of UV compared with contralateral hand on postoperative wrist function. The second critique maybe the patients came from just one single centre, multicentre and more cases studies are needed.

Therefore, the severity of ulnar variance compared with contralateral hand plays an important role in the prognosis of wrist joint function in patients with distal radius fracture. The surgeons should consider it as an important step during preoperative plan.

## Data Availability

All data generated or analysed during this study are included in this published article and are available from the corresponding author on reasonable request.

## References

[1] Tosti R, Foroohar A, Park MJ, Steinberg DR, Bozentka DJ (2011). Distal radius fractures. A review and update. Minerva Ortopedica E Traumatologica..

[2] Kvernmo HD, Krukhaug Y (2013). Treatment of distal radius fractures. Tidsskrift for Den Norske Lægeforening Tidsskrift for Praktisk Medicin Ny Række..

[3] Alluri RK, Hill JR, Ghiassi A (2016). Distal Radius Fractures: Approaches, Indications, and Techniques. Journal of Hand Surgery..

[4] Graham TJ (1997). Surgical correction of malunited fractures of the distal radius. J Amer Acad Orthop Surg..

[5] Palmer AK (1987). The distal radioulnar joint. Anatomy, biomechanics, and triangular fibrocartilage complex abnormalities. Hand Clin..

[6] Bu J, Patterson RM, Morris R, Yang J, Viegas SF (2006). The effect of radial shortening on wrist joint mechanics in cadaver specimens with inherent differences in ulnar variance. J Hand Surg..

[7] Tomaino MM (1998). Ulnar impaction syndrome in the ulnar negative and neutral wrist: diagnosis and pathoanatomy. J Hand Surg Br..

[8] Fernandez DL (2001). Distal radius fracture: the rationale of a classification. Chir Main..

[9] Orbay JL, Fernandez DL (2004). Volar Fixed-Angle Plate Fixation for Unstable Distal Radius Fractures in the Elderly Patient. Journal of hand surgery..

[10] Saffar, P. H. & Pigeau, I. Radiographic imaging of the hand, wrist, and elbow. In: Berger RA, Weiss APC. *Hand Surgery*. Philadelphia: Lippincott Williams and Wilkins. 81–103 (2004).

[11] Dijkstra PU, De Bont LG, Van LT, Boering G (1994). Joint mobility measurements: reliability of a standardized method. Cranio the Journal of Craniomandibular Practice..

[12] Stubbs DF (1979). Visual analogue scale. Br J Clin Pharmacol..

[13] Atroshi L (2000). The disablilities of the arm, shoulder, and hand (DASH) outcome questionnaire: Reliability and validity of the Swedish version evaluated in 176 patients. Acta Orthopaedica Scandinavica.

[14] Mackenney PJ, McQueen MM, Elton R (2006). Prediction of instability in distal radial fractures. J Bone Joint Surg Am.

[15] Steffen L, Marion MF, Thomas P, Prommersberger KJ, Van SJ (2014). Ulnar shortening osteotomy for malunited distal radius fractures:results of a 7-year follow-up with special regard to the grade of radius displacement and post-operative ulnar variance. Archives of Orthopaedic and trauma Surgery..

[16] Tatebe M, Nakamura R, Horii E, Nakao E (2005). Results of ulnar shortening osteotomy for ulnocarpal impaction syndrome in wrists with neutral of negative ulnar variance. J Hand Surg [Br]..

[17] Nathan PA, Meadows KD (1987). Ulna-minus variance and Kienböck’s disease. J Hand Surg..

[18] Nakamura R, Tanaka Y, Imaeda T, Miura T (1991). The influence of age and sex on ulnar variance. J Hand Surg [Br]..

[19] Yoshida T (1990). Aged-onset Kienböck’s disease. Arch Orthop Trauma Surg..

[20] Gelberman RH, Salamon PB, Jurist JM, Posch JL (1975). Ulnar variance in Kienböck’s disease. J Bone Joint Surg Am..

[21] Tahririan MA, Javdan M, Nouraei MH, Dehghani M (2013). Evaluation of instability factors in distal radius fractures. J Res Med Sci..

[22] Stahl S (2013). Critical analysis of causality between negative ulnar variance and kienbock disease. Plast Reconstr Surg..

[23] Goeminne S, Degreef L, De SL (2010). Negative ulnar variance has prognostic value in progression of kienbock disease. Acta Orthop Belg..

[24] Kodama N, Takemura Y, Ueba H, Imai S, Matsusue Y (2014). Acceptable parameters for alignment of distal radius fracture with conservative treatment in elderly patients. J Orthop Sci..

[25] Brennan SA (2016). Volar plate versus k-wire fixation of distal radius fractures. Injury-international Journal of the Care of the Injured..

[26] Dario P (2014). Is it really necessary to restore radial anatomic parameters after distal radius fractures?. Injury-international Journal of the Care of the Injured..

